# Effects of Acute Caffeine Supplementation on Performance and Physiological Responses in CrossFit: A Systematic Review and Three-Level Meta-Analysis

**DOI:** 10.3390/nu18182969

**Published:** 2026-09-10

**Authors:** Jishu Xiao, Jinlong Zheng, Qixin Lin, Xin Wang, Linyin Xu, Yanzheng Xie, Yixiang Peng, Yun Gong, Yinhang Cao

**Affiliations:** 1 Department of Public Physical Education, Fujian Agriculture and Forestry University, Fuzhou 350002, China; xiaojishu319000@163.com; 2 School of Athletic Performance, Shanghai University of Sport, Shanghai 200438, China; 2521811013@sus.edu.cn (J.Z.); 22100815@sus.edu.cn (Q.L.); caoyinhang@sus.edu.cn (Y.C.); 3 China Institute of Sport and Health Science, Beijing Sport University, Beijing 100084, China; wangxin7.30@outlook.com; 4 Faculty for Physical Education, Shanghai International Study University, Shanghai 200083, China; 02637@shisu.edu.cn; 5 School of Physical Education, Hunan University, Changsha 410082, China; 15571605718@163.com; 6 Faculty of Health Sciences and Sports, Macao Polytechnic University, Macao 999078, China; p2525351@mpu.edu.mo; 7 School of Physical Education, Shanghai University of Sport, Shanghai 200438, China

**Keywords:** ergogenic aids, caffeine, CrossFit, high-intensity functional exercise, physiological indicators

## Abstract

**Objectives**: We quantified the influence of acute caffeine (CAF) supplementation on CrossFit performance and physiological outcomes and examined potential effect modifiers. **Methods**: Randomized crossover studies involving CAF supplementation in CrossFit were identified through systematic searches of multiple electronic databases. Performance effect sizes were expressed as Hedges’ *g* and pooled within a three-level random-effects framework, while physiological outcomes were synthesized using conventional two-level models. Effect modification was examined across training status (recreationally active vs. trained) and CAF delivery form (capsule vs. beverage). Meta-regression was additionally used to assess whether relative CAF dose was associated with changes in CrossFit performance. **Results**: The final synthesis comprised seven studies with 103 participants, including 13 women. A small favorable effect of CAF was found for overall CrossFit performance (*g* = 0.13, 95% CI: 0.05–0.22), accompanied by a higher blood lactate concentration (*g* = 0.53, 95% CI: 0.23–0.82). Stratified estimates favored CAF in recreationally active participants (*g* = 0.20, 95% CI: 0.06–0.34) and when administered in capsule form (*g* = 0.17, 95% CI: 0.06–0.29); nevertheless, evidence for between-subgroup differences was absent (all *p* subgroup > 0.05). The relative CAF dose did not explain variation in performance effects in either the linear (*β*_1_ = 0.04, *p* = 0.570) or nonlinear model (*β*_2_ = −0.004, *p* = 0.578). **Conclusions**: The available evidence suggests a small ergogenic benefit of acute CAF supplementation for selected CrossFit performance outcomes, accompanied by elevated blood lactate concentrations. Subgroup analyses revealed no significant moderating effect of training status and supplementation protocols. However, the limited number of studies and predominance of male participants warrant cautious interpretation. Future research should prioritize larger randomized controlled trials, greater representation of female athletes, and more standardized CrossFit performance protocols.

## 1. Introduction

CrossFit combines multiple exercise modalities, including powerlifting, Olympic weightlifting, and running, typically performed at high intensity with brief recovery intervals [1,2]. Its training structure integrates aerobic and resistance-based components, with compound movements forming a central part of the program [3]. As one of the most well-known methods [4,5], CrossFit is adaptable to individuals of all fitness levels and can improve cardiovascular endurance and strength [6,7]. However, this high effort during the sessions may elicit metabolic stress and fatigue [8,9,10]. Unlike traditional high-intensity exercise, CrossFit imposes unique physiological demands by concurrently taxing both aerobic and anaerobic energy pathways alongside substantial neuromuscular fatigue. Therefore, there is growing interest in evidence-based nutritional supplementation strategies to attenuate these responses and improve CrossFit performance.

Caffeine (CAF) is frequently used by athletes as a nutritional aid across a broad range of sporting disciplines [11]. It is well-established that CAF exerts ergogenic effects on endurance [12,13] and strength performance [14,15]. As these exercises are key components of CrossFit, CAF may enhance CrossFit performance. Despite mechanistic evidence supporting its potential benefits, findings from trials remain inconsistent. The ergogenic effects of CAF appear to vary depending on participant characteristics and supplementation protocols, with considerable uncertainty surrounding the effects of CAF ingestion due to the lack of consolidated evidence.

To date, Dos Santos Quaresma et al. [16] qualitatively synthesized the impact of CAF on CrossFit capacity, confirming positive trends; however, their work lacked quantitative statistical pooling. Conversely, a subsequent meta-analysis by Martinho et al. [17] performed a quantitative synthesis but found no meaningful improvement in CrossFit performance following CAF ingestion. Interpretation of these findings is constrained by several methodological issues, including the use of outcomes with limited specificity to CrossFit (e.g., 1RM), the absence of moderator analyses addressing training status and CAF dose, and the lack of pooled physiological outcomes. Moreover, three relevant studies [18,19,20] were not incorporated into the previous quantitative review. Collectively, these considerations provide a rationale for a new meta-analytic evaluation that incorporates the broader evidence base and examines both performance and physiological outcomes.

Although earlier quantitative reviews estimated the general ergogenic effect of CAF [16,17], whether this effect varies according to participant or supplementation characteristics remains insufficiently explored. Such variation has practical relevance for tailoring CAF use to individual needs. Evidence from primary studies suggests that responses to CAF may differ with training background and ingested dose, with trained individuals and doses of 4–6 mg/kg showing more favorable performance responses in some studies [21,22]. Accordingly, the present analysis investigated training status and CAF dose as potential sources of variation in the observed effects.

Building upon previous research, the present study advances the existing evidence base by applying a three-level meta-analytic framework to evaluate the effects of acute CAF supplementation on CrossFit performance and physiological outcomes. By accounting for statistically dependent effect sizes derived from the same participant samples, this approach avoids treating correlated outcomes as independent observations and enables more appropriate estimation of the overall effect and its variability. We further examine potential moderators and dose–response patterns to determine whether CAF effects vary across supplementation strategies and study characteristics. Clarifying these patterns may help athletes, coaches, and sports nutrition practitioners make more informed decisions about CAF strategies while balancing potential performance benefits against unnecessary exposure and dose-related adverse effects.

## 2. Methods

The review was registered with PROSPERO under the identifier CRD420261348602. Its conduct and reporting were guided by the PRISMA 2020 recommendations [23].

### 2.1. Search Strategy

To retrieve potentially eligible records available from database inception through 4 August 2026, searches were conducted across PubMed, Web of Science, Scopus, and the Cochrane Library. Search strategies combined controlled vocabulary with free-text terms using Boolean logic, with the complete syntax provided in Supplementary File S1. Coverage was broadened by examining PROSPERO for ongoing review protocols and manually screening the bibliographies of relevant reviews for eligible studies that might have been missed during database retrieval.

### 2.2. Study Eligibility

Pre-established criteria for inclusion and exclusion were applied to determine study eligibility using the PICOS framework: (1) healthy adult participants (aged 18 years or above) with no restrictions on training status; (2) an experimental condition involving ingestion of a CAF-containing substance before and/or during CrossFit; (3) a placebo-control condition; (4) at least one outcome of overall CrossFit performance (peak power output [PPO], mean power output [MPO], and number of repetitions completed) as well as physiological outcomes (e.g., heart rate [HR], blood lactate levels [BLC], and ratings of perceived exertion [RPE]); and (5) double-blind randomized crossover trials.

Exclusion criteria: (1) not published in English; (2) CAF on long-term CrossFit interventions; (3) non-peer-reviewed literature (e.g., preprints and grey literature), unpublished studies, and conference abstracts, although peer-reviewed ahead-of-print articles were eligible for inclusion; (4) research conducted on animals; (5) studies investigating disease treatment and cure effects.

### 2.3. Selection Process

Initial deduplication of the retrieved records was conducted by one investigator (J.X.) using EndNote X9 (Clarivate Analytics, Philadelphia, PA, USA). The deduplicated records were subsequently cross-checked for residual duplicates by J.X. and J.Z. during independent title and abstract screening, with titles, authors, publication years, and DOIs used to identify potentially missed duplicates. J.X. and J.Z. then independently assessed the titles and abstracts of the remaining records. Eligibility disagreements were referred to a third investigator (Q.L.) for resolution. Articles retained after preliminary screening were independently assessed in full text by J.X. and J.Z. Any disagreements were resolved through discussion based on the prespecified eligibility criteria.

### 2.4. Assessment of Methodological Quality and Risk of Bias

We applied the Cochrane Risk of Bias 2 (RoB 2) tool to assess risk of bias in the eligible crossover trials [24]. Potential bias was screened across six core domains: (1) random sequence generation, (2) period/carryover effects, (3) intervention fidelity, (4) outcome data availability, (5) outcome assessment methods, and (6) reporting choices. Two assessors (J.X. and J.Z.) completed these appraisals independently. Any divergent evaluations were settled via mutual consultation or escalated to a third author (Q.L.).

### 2.5. Data Abstraction and Coding

J.X. and J.Z. independently compiled the required information in Excel (Version 16.93, Microsoft, Redmond, WA, USA). The recorded information covered author-related details, participant profiles (e.g., sample size, sex, and training status), exercise protocols, CrossFit performance measures (e.g., MPO, PPO, and total work), and physiological variables (e.g., HR, BLC, and RPE). Differences between the two extraction records were reconciled through consensus. Numerical values reported exclusively in graphical form were digitized with WebPlotDigitizer 4.7 [25]. For outcomes measured repeatedly at multiple time points within the same experimental condition, values were averaged to obtain a single summary measure for that outcome; distinct outcomes, performance measures, exercise conditions, and CAF doses were not combined. For results reported with a standard error (SE), the corresponding standard deviation (SD) was derived in accordance with Cochrane recommendations [26].

### 2.6. Statistical Analyses

#### 2.6.1. Calculation of Effect Size and Variance

The magnitude of the CAF-related effect on CrossFit outcomes was determined relative to the placebo condition. For each comparison, the mean difference (MD) was derived from change scores, with the corresponding standard deviation (SD) estimated according to the procedures specified in the Cochrane Handbook (Version 6.5, 2024) [26]:

(1) $$MD=M_{CAF}-M_{PLA}$$

Then the ${SD}_{pooled}$ for crossover experiment studies was calculated as follows [27]:

(2) $${SD}_{pooled}=\sqrt{\frac{{SD}_{CAF}^{2}+{SD}_{PLA}^{2}}{2}}$$

Given that the evidence bases predominantly comprised studies with limited sample sizes, Hedges’ *g* was selected as the summary effect metric because it incorporates a correction for small-sample bias. Within the crossover framework, *g* is formulated as [28]:

(3) $$\mathrm{Hedge}’s\;g=\frac{M_{CAF}-M_{PLA}}{{SD}_{pooled}}\times (1-\frac{3}{4(N-1)-1})$$

To quantify the sampling variance (SE) of *g* under repeated-measures conditions, Equation (4) was applied [28]:

(4) $$\mathrm{SE}=\sqrt{\frac{2\left(1-r\right)}{N}+\frac{g^{2}}{2N}}$$

Here, r denotes the correlation of repeated measurements obtained from the same participants under CAF and placebo conditions. Because this correlation was unavailable in the included reports, a value of 0.50 was specified for the main analysis, consistent with recommendations in the Cochrane Handbook [26]. To examine whether the findings depended on this assumption, the analyses were re-executed using r values of 0.20 and 0.80, and corresponding variations in the pooled performance estimates were evaluated.

#### 2.6.2. Meta-Analysis and Heterogeneity

Physiological outcomes were synthesized within a conventional two-level meta-analytic framework implemented in R (version 4.2.0; R Core Team, Vienna, Austria) using the meta and metafor packages [29]. A random-effects specification was adopted, with standardized mean differences expressed as Hedges’ g. Model parameters were obtained through restricted maximum likelihood (REML) estimation. Effect magnitudes were interpreted using the following thresholds: <0.20, trivial; 0.20 to <0.50, small; 0.50 to <0.80, moderate; and ≥0.80, large [26].

Exercise-performance data required a different analytical structure because individual studies frequently contributed more than one dependent estimate, such as MPO and PPO. Accordingly, these outcomes were synthesized with a three-level model based on the framework described by Assink and Wibbelink [30], thereby accommodating dependence among estimates originating from the same study and avoiding inappropriate treatment of such estimates as independent observations [31]. This structure retains all eligible estimates rather than reducing each study to a single value, allowing the available information to contribute to the pooled analysis [30]. Compared with a conventional random-effects meta-analysis, which generally assumes independent effect-size estimates, the three-level approach explicitly accounts for the dependency of multiple estimates reported within the same study. Total variability was partitioned across three components: uncertainty attributable to effect-size sampling at Level 1, variation among estimates from the same study at Level 2, and variation across studies at Level 3. In this way, the hierarchical dependence of multiple estimates was explicitly incorporated into the model [32]. REML estimation was used for the primary three-level analysis, while maximum likelihood (ML) estimation served as an additional check on the resulting model estimates.

We estimated individual coefficients and their associated 95% confidence intervals (95% CIs) using a t-distribution. Given our application of a random-effects framework, prediction intervals (PIs) were also calculated via the t-distribution to offer supplementary insights beyond traditional 95% CIs [33]. Study heterogeneity was evaluated through both *I*^2^ statistics and PIs. Specifically, *I*^2^ estimates were classified into four tiers: low (0–25%), moderate (25–50%), substantial (51–75%), or considerable (76–100%) [34]. Results with *p* values below 0.05 were considered statistically significant.

To investigate between-study variance and identify potential moderating variables, we performed subgroup analyses and meta-regressions targeting continuous parameters [35]. Subgroup evaluations were executed only when a minimum of 3 eligible studies were available [26], whereas meta-regressions required at least 10 studies per predictor [36]. Because only seven independent studies (k = 7) were available, the dose–response meta-regression was conducted solely as an exploratory, hypothesis-generating analysis rather than for confirmatory inference. The resulting coefficients were therefore interpreted cautiously. The categorical variables examined in subgroup analyses comprised: (a) participant training status (recreationally active vs. trained) and (b) CAF administration form (capsule vs. beverage).

Participants’ training status was stratified into recreationally active and trained tiers in accordance with prior frameworks [37]. Model selection for trajectory shapes (linear vs. quadratic) relied on achieving the lowest bias-corrected Akaike information criterion [38]. We implemented all analytical procedures in metafor and constructed visual representations using ggplot2 [39]. Additionally, statistical power across pooled effects and subgroup tiers was evaluated using the metameta package as descriptive indicators of statistical sensitivity [40]. To harmonize dosage metrics, absolute intake values were standardized to relative doses (mg/kg) using the mean body mass of each study sample, as calculated below:

(5) $$\mathrm{Relative}\;\mathrm{dose}\;\left(\mathrm{mg}/\mathrm{kg}\right)=\frac{\mathrm{Absolute}\;\mathrm{dose}\;\left(\mathrm{mg}\right)}{\mathrm{Mean}\;\mathrm{body}\;\mathrm{mass}\;\left(\mathrm{kg}\right)}$$

#### 2.6.3. Assessment of Reporting Bias and Result Stability

To assess potential publication bias, funnel plots [41] were visually inspected and Egger’s regression test [42] was conducted for analyses including more than 10 studies (k > 10) [43]. A non-significant Egger’s test (*p* > 0.05) was interpreted as indicating no statistical evidence of funnel-plot asymmetry. The robustness of the pooled effect estimates from both the two-level and three-level models was examined using sequential leave-one-out sensitivity analyses. For the three-level models, potentially influential observations at Levels 2 and 3 were identified using studentized residuals [44], Cook’s distance [45], and hat values. Observations were considered potentially influential if their Cook’s distance or hat value exceeded three times the corresponding mean value, or if the absolute studentized residual exceeded 3. The three-level models were then re-estimated after excluding the identified influential observations to assess the robustness of the results. Finally, given the small number of independent clusters (k = 7), the three-level model results were additionally examined using cluster-robust variance estimation with a CR2 small-sample adjustment and Satterthwaite degrees-of-freedom approximation [46].

### 2.7. Confidence in the Evidence

We applied the GRADE framework to evaluate confidence in the available evidence [47]. Ratings spanned a four-level scale from very low to high. Initial ratings were performed by one reviewer (J.X.) and subsequently verified by a second reviewer (L.Z.), with any discrepancies resolved through discussion and consensus.

## 3. Results

### 3.1. Identification and Inclusion of Studies

**Figure 1:**
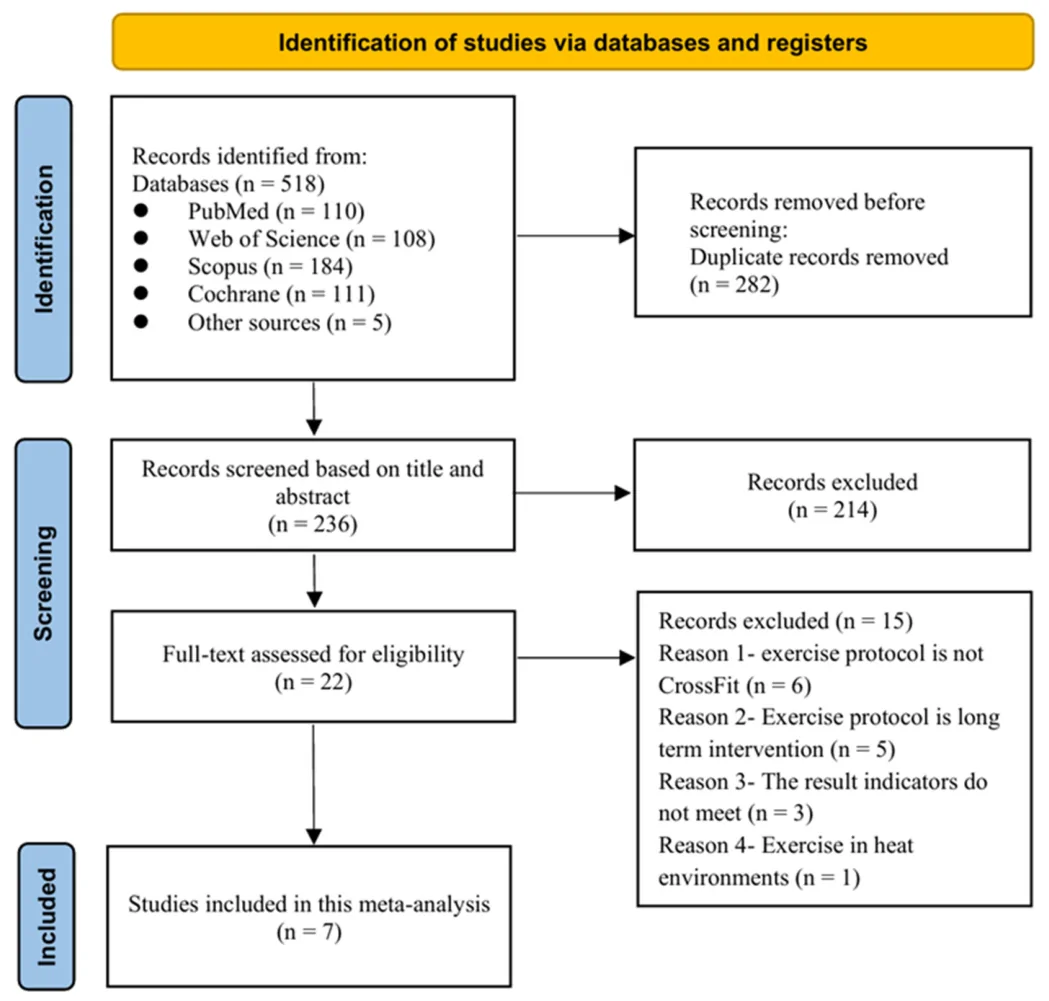
Study identification and inclusion process.

Database searches retrieved 513 records, with five additional records identified through hand searching, resulting in 518 records overall. After applying the predefined eligibility criteria, seven studies were retained for quantitative synthesis [18,19,20,48,49,50,51]. The study identification and selection process is illustrated in Figure 1.

The seven studies collectively enrolled 103 participants, including 90 men (87.4%) and 13 women (12.6%), with enrollment ranging from 8 to 26 per study. Participants refrained from CAF for 24–48 h before testing. CAF doses of approximately 1.4–9 mg/kg were delivered either in capsules or beverages. Both recreationally active and trained populations were represented. Across the included studies, CrossFit performance was primarily assessed using repetition-based outcomes and power-based measures, including the number of repetitions completed, mean power output (MPO), and peak power output (PPO). Further details of the study characteristics are summarized in Table 1.

### 3.2. Primary Analysis

**Figure 2:**
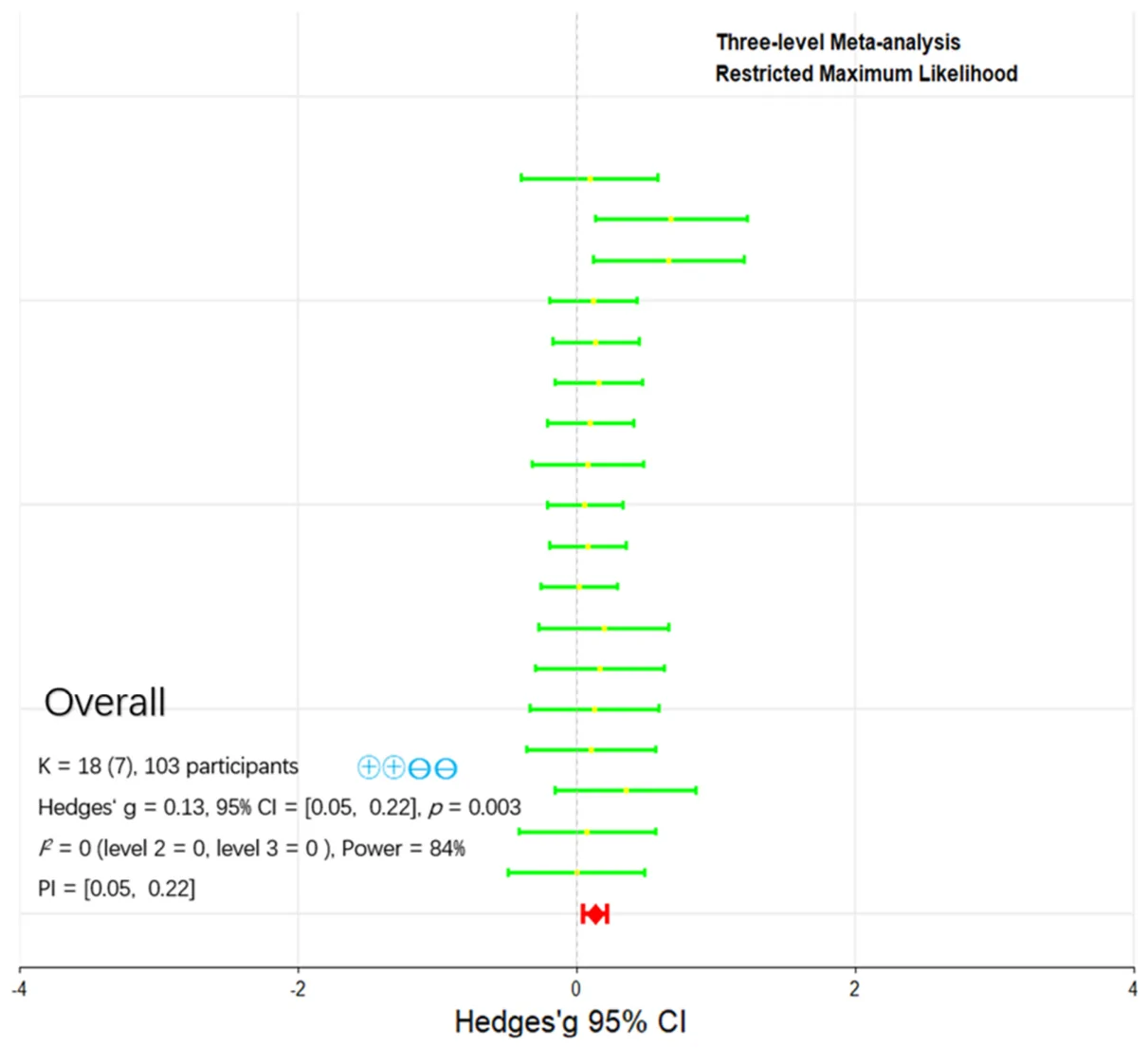
Summary estimates of the effects of acute caffeine intake on overall CrossFit performance. K denotes the number of effect estimates synthesized; Hedges’ *g*, the standardized effect estimate; CI, confidence interval; PI, prediction interval; *I*^2^, heterogeneity; and Power, statistical power. The *p* value refers to the test of the pooled estimate, and blue circles indicate the GRADE certainty rating. K = 18 effect estimates derived from k = 7 independent study clusters.

Overall CrossFit performance showed a small effect favoring CAF (*g* = 0.13, 95% CI: 0.05–0.22, *p* = 0.003; Figure 2). Variance partitioning indicated no detectable heterogeneity at either Level 2 (0%) or Level 3 (0%), with the observed variability entirely attributable to Level 1 sampling error.

**Figure 3:**
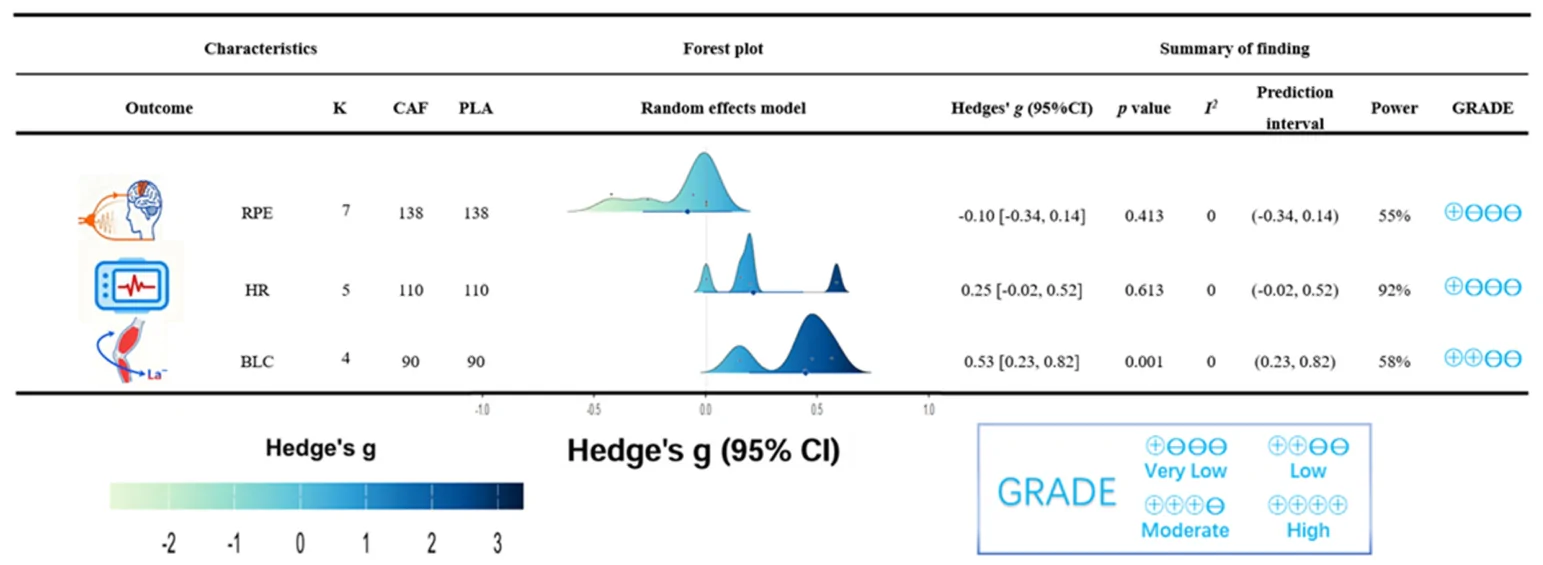
Meta-analytic estimates for physiological and perceptual outcomes under caffeine versus placebo conditions during CrossFit. Effect magnitude is expressed as Hedges’ *g* with a 95% CI, while *I*^2^ describes heterogeneity and K gives the number of estimates entering each synthesis. In the plot table, K = 18 effect estimates derived from k = 7 independent study clusters. The reported *p* values correspond to tests of the pooled effects. CAF and PLA indicate caffeine and placebo conditions, respectively. HR, heart rate; BLC, blood lactate concentration; RPE, rating of perceived exertion.

**Figure 4:**
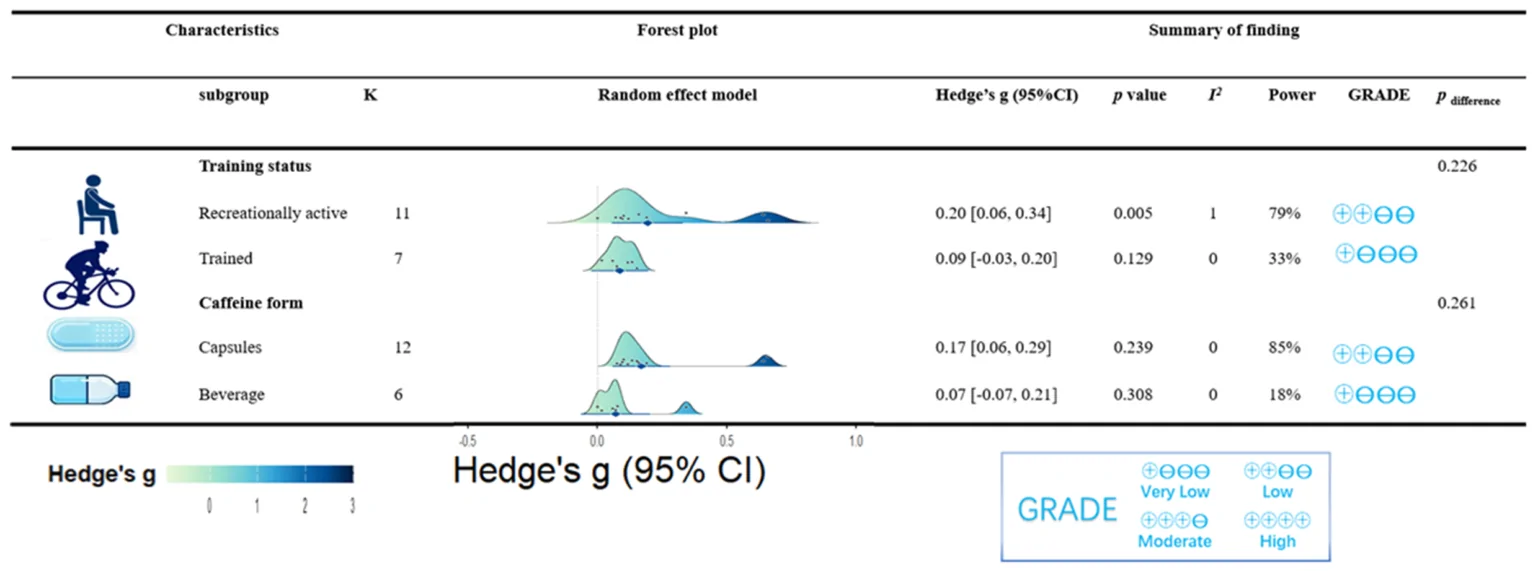
Pooled effects on CrossFit performance were examined separately according to training status and CAF delivery form. Hedges’ *g* and its 95% CI summarize the magnitude and precision of each estimate. *I*^2^ reflects the degree of heterogeneity, while K gives the number of estimates entering the analysis. The overall-effect test is represented by *p*, and *p* difference denotes the test for subgroup differences.

For physiological outcomes, BLC was elevated following CAF administration (*g* = 0.53, 95% CI: 0.23–0.82, *p* = 0.001), whereas HR did not differ significantly between CAF and placebo conditions (*g* = 0.25, 95% CI: −0.05–0.52, *p* = 0.613; Figure 3). Similarly, no statistically significant difference between conditions was detected for RPE (*g* = −0.10, 95% CI: −0.34–0.14, *p* = 0.413; Figure 4).

### 3.3. Moderator Analysis

When stratified by training status, CAF favored performance in recreationally active participants (*g* = 0.20, 95% CI: 0.06–0.34, *p* = 0.005), whereas the estimate for trained participants included the null (*g* = 0.09, 95% CI: −0.03–0.20, *p* = 0.129). The test for subgroup differences did not indicate effect modification by training status (*p* subgroup > 0.05; Figure 4).

A similar stratified analysis was conducted according to the CAF delivery form. The estimate for capsules favored CAF (*g* = 0.17, 95% CI: 0.06–0.29, *p* = 0.003), while the estimate for beverages encompassed the null (*g* = 0.07, 95% CI: −0.07–0.21, *p* = 0.308). No evidence of effect modification by delivery form was identified (*p* subgroup > 0.05; Figure 4).

**Figure 5:**
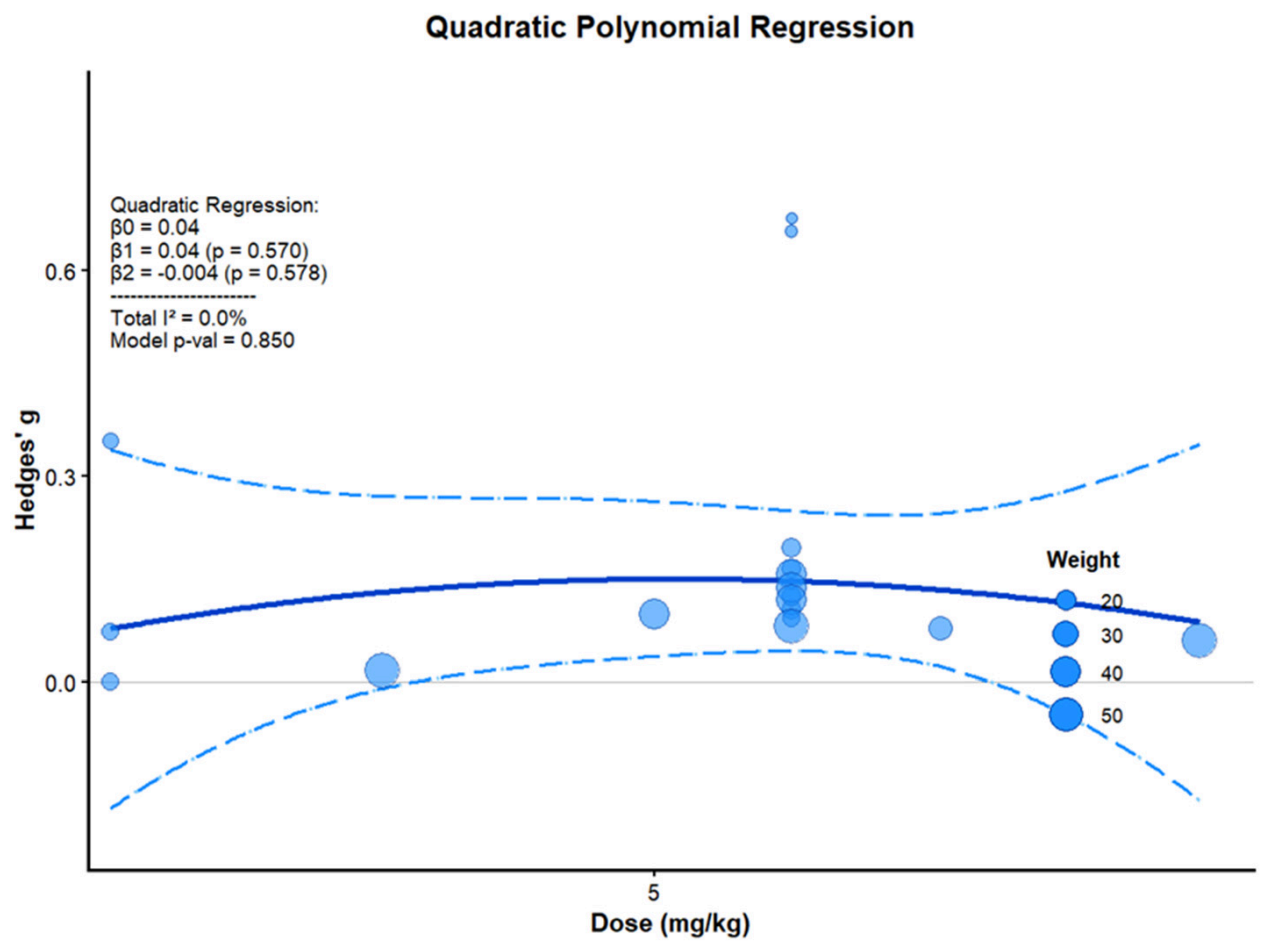
Dose-effect meta-regression plot. *β*_0_, intercept; *β*_1_, *β*_2_, slope coefficients; *I*^2^, heterogeneity;; dotted boundaries, prediction interval; *p*, model significance.

Meta-regression analysis showed that relative CAF dose did not significantly moderate performance outcomes in the linear association (*β*_1_ = 0.04, *p* = 0.570) or for the nonlinear association (*β*_2_ = −0.004, *p* = 0.578; Figure 5).

### 3.4. Quality of Study Methods

**Figure 6:**
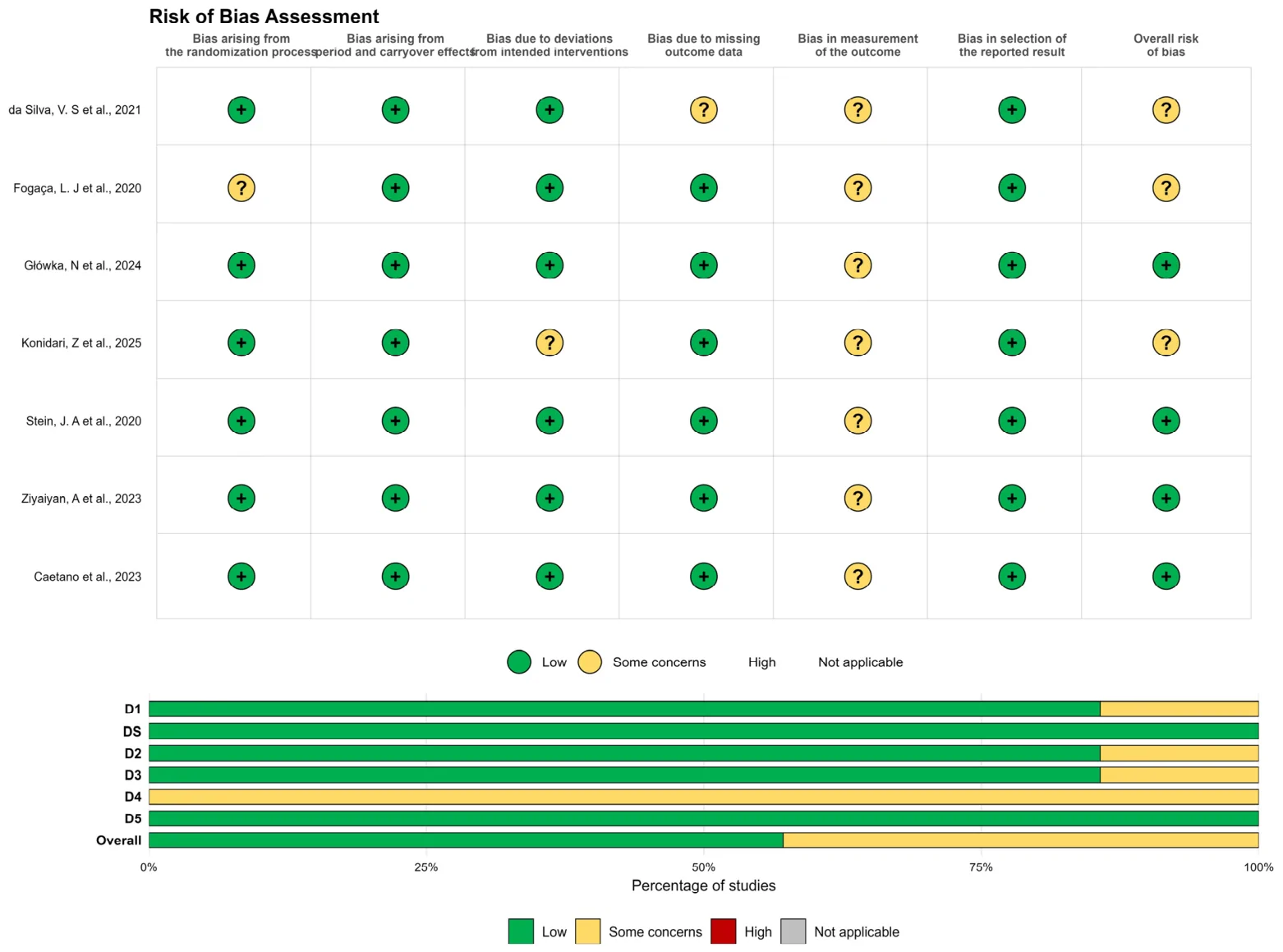
Risk of bias summary: determination included studies [18,19,20,48,49,50,51].

Risk-of-bias judgments were generally favorable across most domains, including randomization, adherence to intended interventions, missing outcome data, period and carryover effects, and selective reporting. Outcome measurement represented the principal source of uncertainty, with all studies receiving a judgment of some concerns in this domain. At the study level, four of the seven studies were classified as low risk, while the remaining three were judged as having some concerns; none were categorized as high risk (Figure 6).

Potential publication bias was examined through funnel-plot inspection and Egger’s regression test. The funnel plot showed no obvious asymmetry, and Egger’s test was non-significant (*p* > 0.05), providing no statistical indication of funnel-plot asymmetry for overall CrossFit performance. Given the small number of independent studies (k = 7), publication bias could not be reliably assessed; therefore, Egger’s test was not performed, and the funnel plot was interpreted descriptively only.

### 3.5. Sensitivity Analysis

#### 3.5.1. Sensitivity Analysis for Primary Effect

**Figure 7:**
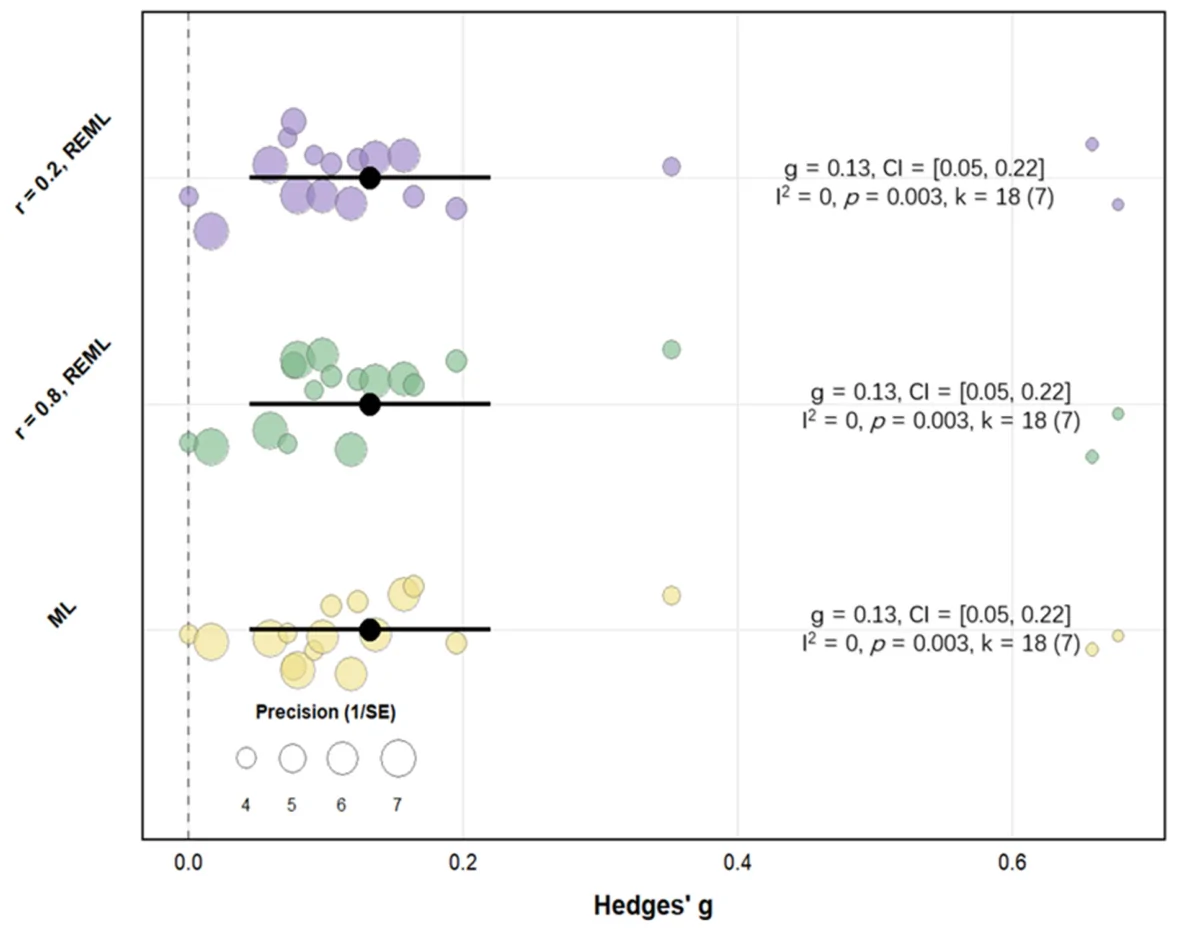
Robustness assessment of the overall effect under alternative analytical specifications. The pooled Hedges’ *g* estimates are presented with their 95% confidence intervals. K denotes the number of effect sizes contributing to the synthesis; *p* represents the significance probability; r refers to the assumed correlation between the CAF and PLA conditions; ML indicates maximum likelihood estimation; REML indicates restricted maximum likelihood estimation; and *I*^2^ describes the proportion of variability attributable to heterogeneity.

To evaluate the stability of the primary effect size, sensitivity analyses were first performed by introducing two distinct within-subject correlation coefficients (r = 0.2 and r = 0.8) for crossover designs. The resulting combined effect size exhibited negligible variation, maintaining statistical significance across all scenarios (*p* < 0.05; Figure 7). Furthermore, varying the within-study correlation assumption caused no alteration in heterogeneity estimates, confirming that the overall conclusions were highly insensitive to these parameter choices. Similar consistency was observed between the ML and REML estimation approaches.

Next, sequential leave-one-out diagnostics were executed across both two-level and three-level meta-analytic frameworks. These procedures demonstrated that excluding the study by Główka et al. [49] altered the pooled estimate for HR from a non-significant result to one that reached statistical significance, implying potential fragility in the HR outcome (Supplementary File S4).

Regarding influential cases, neither the two-level nor the three-level meta-analysis revealed any outlying data points based on Cook’s distance and studentized residual criteria. Additionally, application of a robust variance estimation sensitivity test yielded an identical summary effect estimate (*g* = 0.13, 95% CI 0.02 to 0.25, *p* = 0.032), further reinforcing the stability and validity of the primary three-level model.

#### 3.5.2. Sensitivity Analysis for Moderator Effect

The moderator results were subjected to additional robustness checks in which individual studies were removed sequentially and the corresponding models were refitted. Re-estimation of the three-level models after each exclusion resulted in little variation in the moderator estimates, and none of the omissions materially changed the overall interpretation. This pattern was consistently observed across the moderator models, indicating that the corresponding findings were not substantially dependent on any single study.

## 4. Discussion

To date, this is the first comprehensive review to specifically evaluate how CAF influences CrossFit performance and physiological responses, while also addressing methodological challenges related to the inclusion of multiple effect sizes from individual studies in meta-analytic modeling.

### 4.1. Effect of CAF on CrossFit

Pooled results indicated a statistically significant ergogenic effect of CAF on CrossFit performance relative to placebo (*g* = 0.13, 95% CI: 0.05–0.22) (Figure 3). In contrast, Martinho et al. [17] synthesized evidence from four investigations but found insufficient evidence to support a beneficial effect of CAF on CrossFit performance. The conflicting conclusions could stem from their use of outcome measures that do not directly represent CrossFit-specific performance, such as 1RM, potentially reducing the validity of their conclusions. Furthermore, the data included in the review were not comprehensive. For instance, Fogaça et al. [48] only included upper-limb MPO and PPO, omitting the corresponding lower-limb outcomes. These factors may have reduced the observed effect size. Given that CrossFit is characterized by high-intensity exercise sessions [1], this finding aligns with previous meta-analyses demonstrating that CAF significantly enhances high-intensity exercise performance across laboratory and real-world competitive settings [52,53]. However, no performance benefit of CAF was detected by Lopes-Silva et al. [54] during repeated-sprint exercise. This null response might be related to the exercise design, which consisted of intense sprint bouts shorter than 10 s separated by recovery intervals of less than 60 s [55]. Under these conditions, performance relies heavily on phosphocreatine resynthesis and anaerobic glycolysis [56], potentially limiting the ergogenic efficacy of CAF [57,58]. From a mechanistic perspective, reduced performance during these high-intensity efforts may arise from both central and peripheral factors, including increased adenosine availability and diminished phosphocreatine stores, respectively [56,59,60]. The ergogenic actions of CAF are thought to be mediated, at least partly, through interference with adenosine receptor signaling, with consequences for physiological processes in both the central nervous system and peripheral tissues [61].

At the central level, blockade of adenosine signaling may facilitate cortical activation and delay the development of central fatigue [62,63]. The pooled analysis also identified a higher BLC following CAF intake (*g* = 0.53, 95% CI: 0.23–0.82; Figure 4), accompanying the greater exercise output observed under CAF conditions. This response may partly reflect enhanced sympathetic activity [64]. Accordingly, the ability to attain higher BLC may reflect a greater glycolytic contribution and the capacity to sustain higher exercise intensities during CrossFit. Although the pooled effect on HR did not reach statistical significance, removal of Główka, N et al. [49] in the sensitivity analysis yielded a statistically significant pooled estimate (Supplementary Materials S4).

RPE remained broadly comparable following CAF and placebo ingestion [65], as reflected by the pooled effect estimate (*g* = −0.10, 95% CI: −0.34–0.14; Figure 4), whereas performance outcomes favored CAF during CrossFit exercise. The coexistence of greater physical output with unchanged perceived exertion may indicate altered central processing of fatigue-related sensory feedback [62,63], allowing athletes to perform more work without perceiving the task as more strenuous.

At the peripheral level, CAF may directly influence skeletal muscle function by antagonizing local adenosine receptors [63], which subsequently enhances Na^+^-K^+^ pump activity to preserve membrane excitability [66]. Ultimately, this mechanism could partially offset reductions in excitation–contraction coupling efficiency and support force production during CrossFit-type exercise. Although the small number of included studies may have limited the stability of the variance estimates in the three-level model, consistent findings across the RVE and other sensitivity analyses strengthened confidence in the robustness of the primary results.

Although no within- or between-study heterogeneity was detected, this finding should be interpreted cautiously given the clinical and methodological variability across studies and the limited number of independent studies (k = 7). The zero variance estimates may reflect limited power to detect heterogeneity rather than true homogeneity of effects. Because the estimated variance components were zero, the model-based prediction interval coincided with the confidence interval; this apparent precision should likewise be interpreted cautiously.

### 4.2. Potential Moderators of Supplementation Protocols

#### 4.2.1. CAF Dose

Variation in relative CAF intake did not explain performance differences in either the linear (*β*_1_ = 0.04, *p* = 0.570) or nonlinear (*β*_2_ = −0.004, *p* = 0.578) meta-regression model (Figure 6). Although the present data do not statistically confirm a dose–response relationship, descriptive inspection of the included trials suggests a possible inverted U-shaped pattern, wherein moderate doses of CAF (4–6 mg/kg) appear to confer the most favorable ergogenic effects. This observation closely aligns with previous meta-analytical evidence [61] establishing that an intake range of 4 to 6 mg/kg represents the most efficacious supplementation strategy for exercise performance. Safety outcomes were not formally synthesized in the present meta-analysis. Descriptively, one included study using a high CAF dose (7–9 mg/kg) reported tachycardia or palpitations (17%), dizziness (25%), and gastrointestinal distress (33%) [19]. Previous reviews have similarly reported a greater frequency of adverse events at higher CAF doses [67,68]. However, because these safety data were derived from individual studies and external literature rather than a quantitative synthesis in the present review, no dose-dependent safety conclusions can be drawn. Likewise, although lower CAF doses may theoretically reduce the risk of adverse effects, the current evidence is insufficient to determine whether their ergogenic effects differ systematically from those of higher doses [69,70,71,72].

Although numerically larger effects were observed at moderate CAF doses (4–6 mg/kg), neither the linear nor the nonlinear meta-regression identified a statistically significant dose–response relationship. Accordingly, caution is warranted when drawing dose-related conclusions, as the current evidence does not support identifying a particular CAF dose as optimal. Future high-quality, large-scale trials are warranted to explicitly validate this inverted U-shaped dose–response hypothesis in CrossFit protocol.

#### 4.2.2. CAF Form

No evidence was found that the ergogenic response varied according to the form in which CAF was administered (subgroup *p* > 0.05). When examined separately, capsule administration yielded a pooled effect estimate of *g* = 0.17 (95% CI: 0.06–0.29), whereas the estimate for CAF delivered as a beverage was *g* = 0.07 (95% CI: −0.07–0.21) (Figure 5). This observed subgroup pattern is broadly consistent with previous meta-analyses suggesting that capsule delivery may yield more consistent ergogenic benefits [61,68]. Given that the beverage subgroup comprised only two independent studies, the analysis had limited capacity to detect effect modification by administration form. Accordingly, the nonsignificant subgroup test should be interpreted as insufficient evidence of effect modification rather than evidence that administration form has no influence on the ergogenic effect of CAF.

### 4.3. Potential Moderators of Training Status

No significant moderating effect of training status was detected (*p* for subgroup difference > 0.05), indicating insufficient evidence that the effect of CAF differed between recreationally active and trained athletes. Although the pooled estimate was significant among recreationally active athletes (*g* = 0.20, 95% CI: 0.06–0.34) but not among trained athletes (*g* = 0.09, 95% CI: −0.03–0.20), this descriptive pattern should not be interpreted as evidence of a true subgroup difference. Previous evidence has suggested that training status may influence responsiveness to CAF [73,74], potentially because highly trained athletes have less scope for further performance gains [12,75]. However, given the nonsignificant subgroup interaction and the small number of independent studies, this explanation remains speculative and requires confirmation in adequately powered studies.

### 4.4. Application of the Findings

The available evidence suggests a small ergogenic effect of acute CAF intake on CrossFit performance. Although even small performance gains could be relevant in competitive settings where rankings or qualification outcomes are determined by narrow margins, the practical significance of this effect remains uncertain. Given the heterogeneity of the performance outcomes included, the standardized effect cannot be reliably translated into specific sport-related gains, such as additional repetitions in AMRAP workouts or seconds saved during timed benchmark WODs. Furthermore, the limited evidence base (k = 7 independent studies) does not permit reliable characterization of the dose–response relationship or identification of an optimal CAF dose.

### 4.5. Limitations and Future Considerations

Although the review was conducted in accordance with PRISMA guidelines, its findings need to be interpreted in light of several methodological constraints. First, the evidence base was limited to seven independent studies (103 participants), reducing the precision of pooled estimates and the reliability of subgroup, dose–response, and publication-bias assessments. Some subgroups were particularly sparse; for example, only two studies examined beverage-based CAF, precluding reliable comparisons by administration form. Second, women represented only 12.6% of the pooled sample. Given that CAF metabolism and responses may be influenced by sex-related hormonal factors and oral contraceptive use [76], females comprised only 12.6% of the overall sample. Thus, these findings predominantly reflect male participants and should be applied to female CrossFit athletes with caution. Third, considerable methodological variability existed across CrossFit protocols and performance outcomes, limiting direct comparability across studies and complicating the practical interpretation of the pooled effect. Additionally, restricting eligibility to English-language, peer-reviewed publications and not systematically searching grey literature sources may have introduced language and publication bias and resulted in the omission of relevant unpublished or non-English evidence [77]. Fourth, habitual CAF intake and prior exposure to comparable doses were inconsistently reported. Because habituation or tolerance may modify responses to acute CAF supplementation, this represents an additional source of uncertainty. Genetic variation was also not considered, although previous evidence suggests that responses to CAF may differ by genotype [13]. In addition, none of the included studies blinded outcome assessors or assessed blinding success. Because CAF can produce noticeable physiological sensations, expectancy and detection bias cannot be excluded, particularly for subjective outcomes such as RPE, although their influence may be smaller for objectively measured performance outcomes. Fifth, whereas the other included comparisons evaluated CAF without unmatched active co-interventions, da Silva et al. [20] administered CAF as part of a multi-ingredient energy drink containing taurine and carbohydrates. Consequently, the effect from this study cannot be attributed exclusively to CAF. However, excluding this study in the leave-one-out sensitivity analysis did not materially alter the pooled estimate, suggesting that its inclusion had limited influence on the overall finding. Finally, given the limited number of independent studies, the meta-regression analyses had limited inferential capacity; therefore, the observed dose–response patterns should be considered exploratory and hypothesis-generating rather than confirmatory.

## 5. Conclusions

The available evidence from seven randomized crossover studies comprising 103 participants suggests that acute CAF supplementation may provide a small ergogenic benefit for selected CrossFit performance outcomes and may increase BLC. However, the limited number of independent studies, heterogeneity of CrossFit protocols and caffeine interventions, underrepresentation of women, and uncertainty surrounding moderator analyses limit the precision, generalizability, and practical interpretation of these findings. The current evidence is insufficient to reliably characterize the dose–response relationship or identify an optimal CAF dose. Larger randomized trials with greater female representation and more standardized CrossFit protocols are needed to confirm these findings.

## Figures and Tables

**Table 1:** Overview of the included studies.

Reference	Participants + Age (Years), Body Mass + Training Status	Habitual CAF Intake (mg/day) + CAF Withdrawal (h)	CAF Dosage (mg/kg)	Timing (min)	CAF Form	CrossFit Protocol	Outcomes
Fogaça et al., 2020 [48]	9 M; 28 ± 2; 79 ± 3; recreationally active	0.46 (0.06–0.63) mg per week; 48	6	60	capsules	Five (80% 1RM snatch from the block with 2 min recovery); 3 × 5 (75% 1RM touch-and-go snatches with 90 s recovery); 3 × 60 s (isometric weighted planks with 90 s recovery); 10 min AMRSP 30 double-under and 15 power snatches (34 kg)	①②
Stein et al., 2020 [50]	20 M; 27 ± 6; 84 ± 10; recreationally active	288.3 ± 287.5; 24	5	60	capsules	20-min AMRAP consisting of 5 pull-ups, 10 push-ups, and 15 air squats per round	③⑥
da Silva et al., 2021 [20]	3 F, 5 M; 27 ± 3; 70 ± 13; recreationally active	NA; 24	~1.4	30	beverage	BBP 10 RM test + BS 12 RM test + AMRAP till exhaustion (Wall Climbing + Wall Ball + Box Jump)	③⑥
Ziyaiyan et al., 2023 [18]	20 M; 22 ± 3; 81 ± 12; trained	≤125 mg; 24	6	50	capsules	20 min AMRAP (5 pull-ups, 10 push-ups, and 15 air squats)	①③④⑥
Caetano et al., 2023 [51]	8 M; 30 ± 7; 82 ± 8; recreationally active	NA; 24	6	60	capsules	1 MR back squat test; 60% 1 MR back squat till exhaustion	②③
Główka et al., 2024 ^a^ [49]	16 M, 10 F; 35 ± 7; 77 ± 17; trained	231 mg; 24	3	70	beverage	Three 5-min AMRSP bouts incorporating wall-ball shots, sumo deadlift high pulls, box jumps, push presses, and rowing, separated by 1-min recovery periods	③④⑤⑥
Główka et al., 2024 ^b^ [49]	16 M, 10 F; 35 ± 7; 77 ± 17; trained	231 mg; 24	6	70	beverage	Three 5-min AMRSP bouts incorporating wall-ball shots, sumo deadlift high pulls, box jumps, push presses, and rowing, separated by 1-min recovery periods	③④⑤⑥
Główka et al., 2024 ^c^ [49]	16 M, 10 F; 35 ± 7; 77 ± 17; trained	231 mg; 24	9	70	beverage	Three 5-min AMRSP bouts incorporating wall-ball shots, sumo deadlift high pulls, box jumps, push presses, and rowing, separated by 1-min recovery periods	③④⑤⑥
Konidari et al., 2025 [19]	12 M; 29 ± 4; 80 ± 8; trained	136 ± 97; 24	7.1	60	capsules	Four 5-exercise AMRAP bouts comprising push-ups, power cleans, front squats, sit-ups, and deadlifts, using 50-s work and 10-s recovery intervals	③④⑤⑥

Superscripts a–c denote separate trials from the same study. Abbreviations: M, male; F, female; AMRAP, as many rounds as possible; NA, not available; BBP, barbell bench press; BS, barbell squat; RM, repetition maximum. Outcome codes: ① mean power output; ② peak power output; ③ total repetitions; ④ heart rate; ⑤ blood lactate concentration; ⑥ rating of perceived exhaustion.

## Data Availability

The original contributions presented in this study are included in the article/Supplementary Material. Further inquiries can be directed to the corresponding author.

## Supplementary Materials

The following supporting information can be downloaded at: https://www.mdpi.com/article/10.3390/nu18182969/s1; Supplementary Material File S1: (Search strategy); Supplementary Material File S2: (PEDro Assessment); Supplementary Material File S3: (GRADE Assessment); Supplementary Material File S4: (A Sensitivity Analysis); Supplementary Material File S5: (Funnel plot); Supplementary File S6: (Manuscript Checklist).
