# Supplementary material for: Effects of Acute Caffeine Supplementation on Performance and Physiological Responses in CrossFit: A Systematic Review and Three-Level Meta-Analysis
Source: Nutrients. 2026 Sep 10;18(18):2969. doi: 10.3390/nu18182969 (PMC13610431; doi:10.3390/nu18182969)
Supplement: Supplementary file 1 [file nutrients-18-02969-s001.zip › nutrients-4517476-supplementary.pdf]

Supplementary Material File S1 (Search strategy)

Data	Query	Results
PubMed	("Caffeine"[Mesh] OR caffeine*[tw] OR caffein[tw] OR coffee*[tw] OR "energy drink*"[tw] OR "energy shot*"[tw] OR "pre-workout*"[tw] OR preworkout*[tw] OR "pre workout*"[tw] OR ergogenic*[tw] OR guarana[tw] OR methylxanthine*[tw]) AND ("CrossFit"[tw] OR "Cross-Fit"[tw] OR "Cross Fit"[tw] OR "high-intensity functional training"[tw] OR "high intensity functional training"[tw] OR HIFT[tw] OR "functional fitness"[tw] OR "functional training"[tw] OR WOD*[tw] OR "workout of the day"[tw] OR AMRAP[tw] OR "circuit-based exercise"[Mesh] OR "circuit training"[tw] OR "circuit weight training"[tw] OR "High-Intensity Interval Training"[Mesh] OR "high intensity interval"[tw] OR HIIT[tw] OR "mixed-modal"[tw] OR "mixed modal"[tw] OR "multimodal training"[tw] OR "multi-modal training"[tw])	108
WOS	TS = (caffeine* OR caffein OR coffee* OR "energy drink*" OR "energy shot*" OR "pre-workout*" OR preworkout* OR "pre workout*" OR ergogenic* OR guarana OR methylxanthine*) AND TS=("CrossFit" OR "Cross-Fit" OR "Cross Fit" OR "high-intensity functional training" OR "high intensity functional training" OR HIFT OR "functional fitness" OR "functional training" OR WOD* OR "workout of the day" OR AMRAP OR "circuit-based exercise" OR "circuit training" OR "circuit weight training" OR "High-Intensity Interval Training" OR "high intensity interval" OR HIIT OR "mixed-modal" OR "mixed modal" OR "multimodal training" OR "multi-modal training")	110
Scopus	TITLE-ABS-KEY (caffeine* OR caffein OR coffee* OR "energy drink*" OR "energy shot*" OR "pre-workout*" OR preworkout* OR "pre workout*" OR ergogenic* OR guarana OR methylxanthine*) AND TITLE-ABS-KEY ("CrossFit" OR "Cross-Fit" OR "Cross Fit" OR "high-intensity functional training" OR "high intensity functional training" OR hift OR "functional fitness" OR "functional training" OR wod* OR "workout of the day" OR amrap OR "circuit-based exercise" OR "circuit training" OR "circuit weight training" OR "High-Intensity Interval Training" OR "high intensity interval" OR hiit OR "mixed-modal" OR "mixed modal" OR "multimodal training" OR "multi-modal training")	184
Cochrane	#1<br>("CrossFit":ti,ab,kw OR "Cross-Fit":ti,ab,kw OR "Cross Fit":ti,ab,kw OR "high-intensity functional training":ti,ab,kw OR "high intensity functional training":ti,ab,kw OR HIFT:ti,ab,kw OR "functional fitness":ti,ab,kw OR "functional training":ti,ab,kw OR WOD*:ti,ab,kw OR "workout of the day":ti,ab,kw OR AMRAP:ti,ab,kw OR [mh "circuit-based exercise"] OR "circuit training":ti,ab,kw OR "circuit weight training":ti,ab,kw OR [mh "High-Intensity Interval Training"] OR "high intensity interval":ti,ab,kw OR HIIT:ti,ab,kw OR "mixed-modal":ti,ab,kw OR "mixed modal":ti,ab,kw OR "multimodal training":ti,ab,kw OR "multi-modal training":ti,ab,kw)<br>#2<br>([mh "Caffeine"] OR caffeine*:ti,ab,kw OR caffein:ti,ab,kw OR coffee*:ti,ab,kw OR "energy drink*":ti,ab,kw OR "energy shot*":ti,ab,kw OR "pre-workout*":ti,ab,kw OR preworkout*:ti,ab,kw OR "pre workout*":ti,ab,kw OR ergogenic*:ti,ab,kw OR guarana:ti,ab,kw OR methylxanthine*:ti,ab,kw)<br>#1 AND #2	111

Supplementary Material File S2 (PEDro Assessment)

	Item 1	Item 2	Item 3	Item 4	Item 5	Item 6	Item 7	Item 8	Item 9	Item 10	Item 11	Total	Rating
Fogaça, L. J et al., 2020	YES	0	1	1	1	1	0	1	1	1	1	8	Good
Stein, J. A et al., 2020	NO	1	1	1	1	1	0	1	1	1	0	8	Good
da Silva, V. S et al., 2021	YES	1	1	0	1	1	0	0	0	0	1	5	Fair
Ziyaiyan, A et al., 2023	YES	1	1	1	1	1	0	1	1	1	1	10	Excellent
Caetano et al., 2023	YES	1	1	1	1	1	0	1	1	1	0	9	Good
Główka, N et al., 2024	YES	1	1	1	1	1	0	1	1	1	1	10	Excellent
Konidari, Z et al., 2025	NO	1	0	1	1	1	0	1	1	1	1	8	Good

Note: "excellent" (10–11 points); "good" (7–9 points); "fair" (5–6 points); and "poor" (0–4 points).

1. eligibility criteria were specified.
2. subjects were randomly allocated to groups (in a crossover study, subjects were randomly allocated an order in which treatments were received)
3. allocation was concealed
4. the groups were similar at baseline regarding the most important prognostic indicators
5. there was blinding of all subjects
6. there was blinding of all therapists who administered the therapy
7. there was blinding of all assessors who measured at least one key outcome
8. measures of at least one key outcome were obtained from more than 85% of the subjects initially allocated to groups
9. all subjects for whom outcome measures were available received the treatment or control condition as allocated or, where this was not the case, data for at least one key outcome was analyzed by "intention to treat"
10. the results of between-group statistical comparisons are reported for at least one key outcome
11. the study provides both point measures and measures of variability for at least one key outcome

Supplementary Material File S3 (GRADE Assessment)

Certainty of Evidence Assessment								
Outcome	K	Risk of Bias	Inconsistency	Indirectness	Imprecision	Others	Hedge's g [95% CI] *	GRADE†
Primary Outcome								
CrossFit performance	K = 18	Not serious	Serious	Not serious	Serious	None	0.13 [0.05, 0.22] *	⊕⊕◯◯ Low
RPE	K = 7	Not serious	Serious	Serious	Serious	None	0.10 [-0.34, 0.14]	⊕◯◯◯ Very low
HR	K = 5	Not serious	Serious	Serious	Serious	None	0.25 [-0.02, 0.52]	⊕◯◯◯ Very low
BLC	K = 4	Not serious	Serious	Not serious	Serious	None	0.53 [0.23, 0.82] *	⊕⊕◯◯ Low
Training status								
Recreationally active	K = 11	Not serious	Not serious	Serious	Serious	None	0.20 [0.03, 0.34] *	⊕⊕◯◯ Low
Trained	K = 7	Not serious	Serious	Serious	Serious	None	0.09 [-0.03, 0.20]	⊕◯◯◯ Very low
Caffeine form								
Capsules	K = 12	Not serious	Not serious	Serious	Serious	None	0.17 [0.06, 0.29] *	⊕⊕◯◯ Low
Beverage	K = 6	Not serious	Serious	Serious	Serious	None	0.07 [-0.07, 0.21]	⊕◯◯◯ Very low

Initial certainty: As all included studies used randomized crossover designs, certainty was initially rated as High in accordance with the GRADE framework.

[a] Inconsistency: Downgraded by one level for serious inconsistency because of clinical and methodological diversity across studies, including differences in CrossFit protocols, caffeine dose/timing, and outcome assessment. The limited number of independent studies was also considered when interpreting the absence of detectable statistical heterogeneity.

[b] Indirectness: Downgraded by one level for serious indirectness/applicability concerns where relevant, particularly because women were markedly underrepresented in the available evidence (13/103, 12.6%), limiting applicability to female CrossFit athletes.

[c] Imprecision: Downgraded by one level for serious imprecision when estimates were based on a small number of independent studies (k) and participants and/or had wide confidence intervals that crossed the null or encompassed materially different effect magnitudes.

Statistically significant pooled effect ($p < 0.05$).

† GRADE certainty of evidence: ⊕⊕⊕⊕, High; ⊕⊕⊕○, Moderate; ⊕⊕○○, Low; ⊕○○○, Very low.

Supplementary Material File S4 (A Sensitivity Analysis)

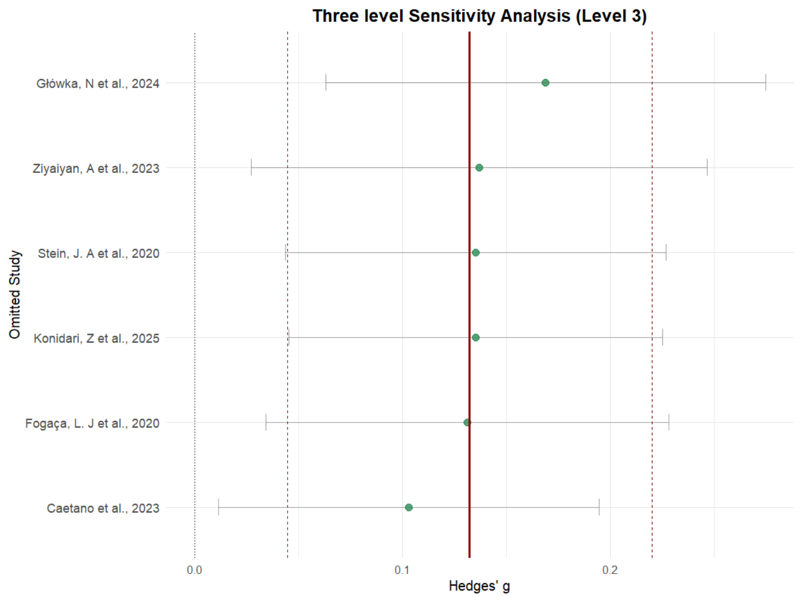

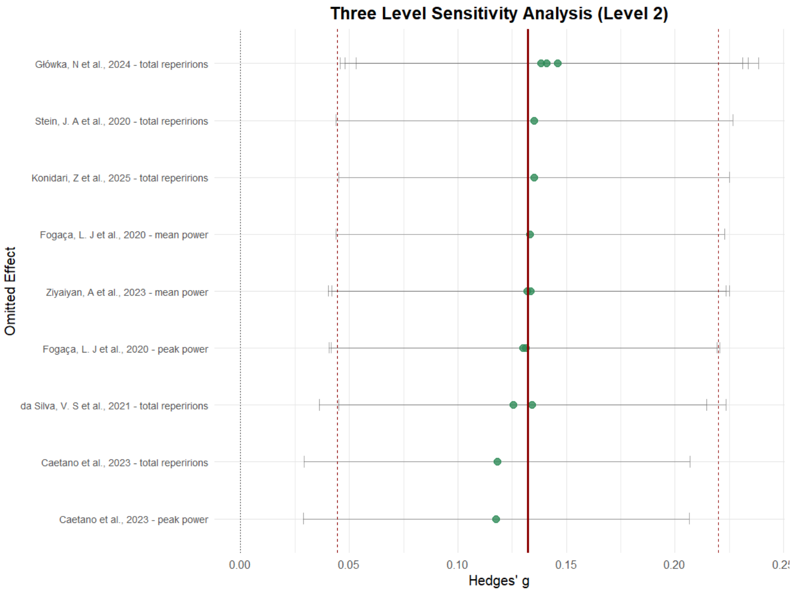

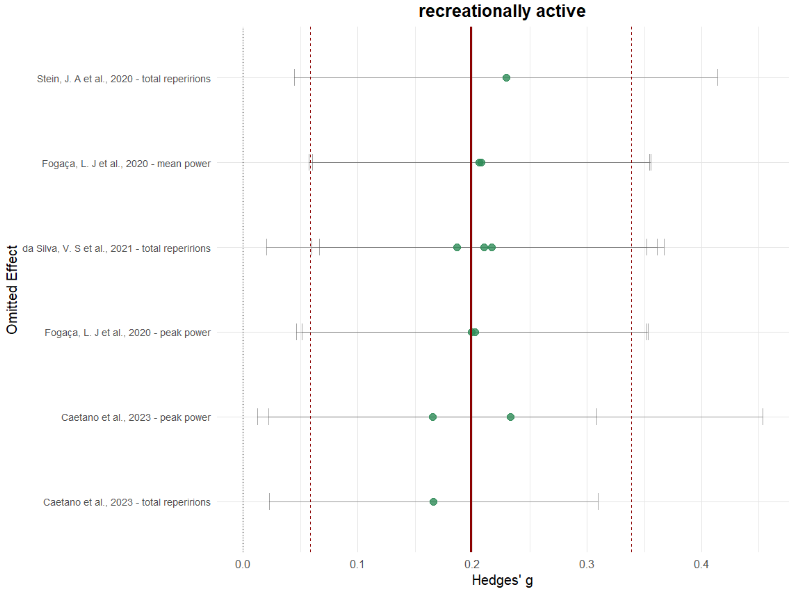

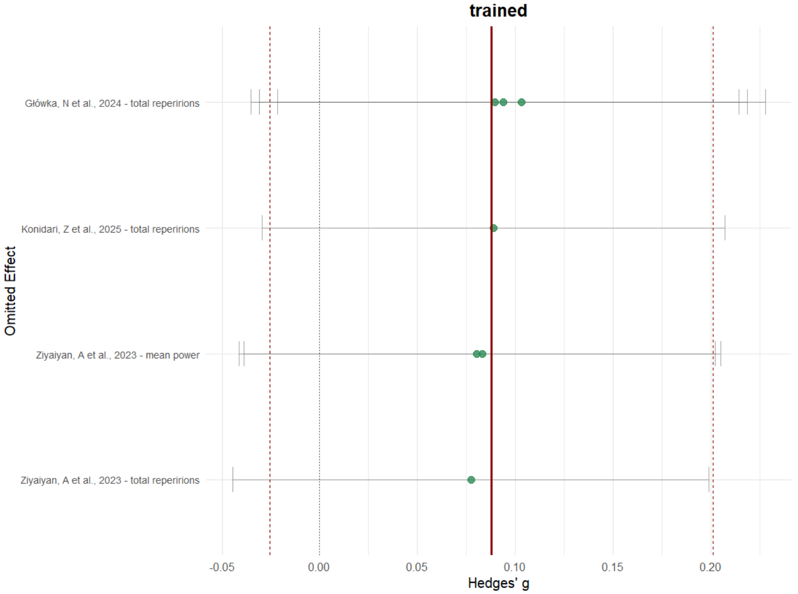

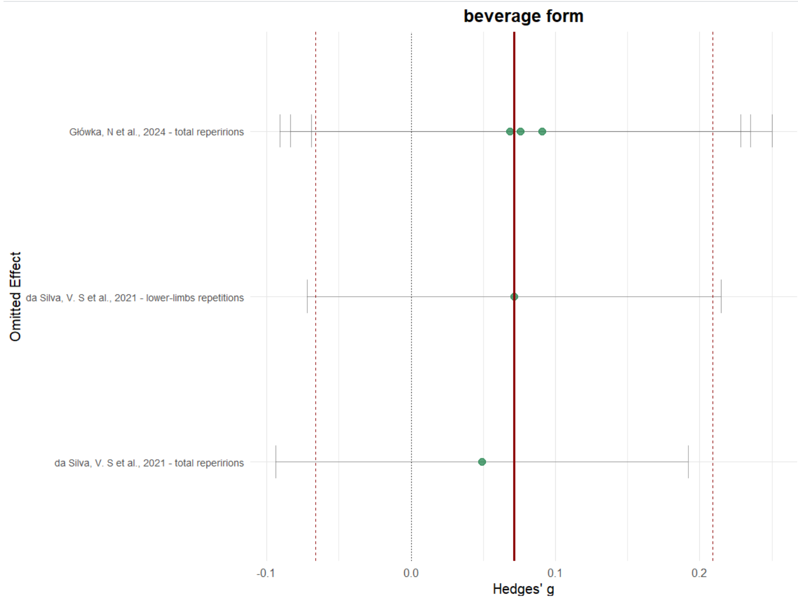
beverage form
Główka, N et al., 2024 - total reperirions
da Silva, V. S et al., 2021 - lower-limbs repetitions
da Silva, V. S et al., 2021 - total reperirions
Omitted Effect
-0.1
0.0
0.1
0.2
Hedges' g

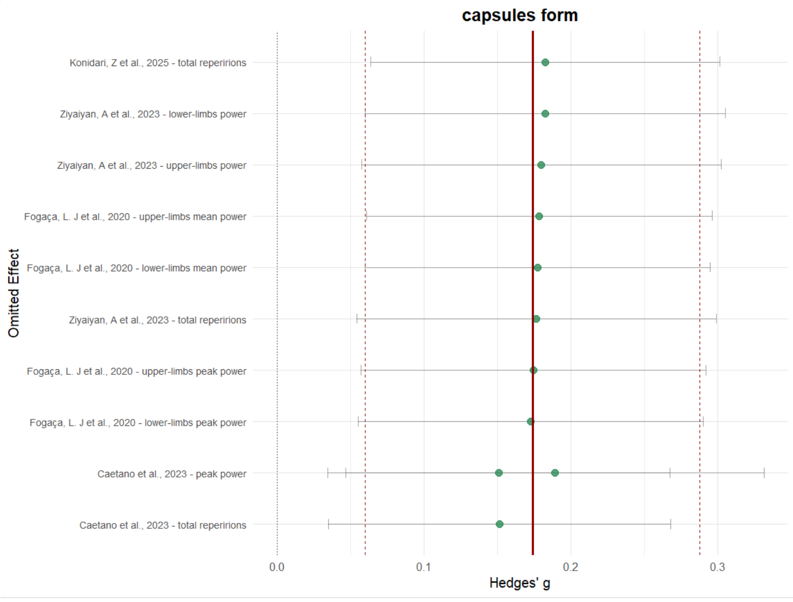
capsules form
Konidari, Z et al., 2025 - total reperirions
Ziyaiyan, A et al., 2023 - lower-limbs power
Ziyaiyan, A et al., 2023 - upper-limbs power
Fogaça, L. J et al., 2020 - upper-limbs mean power
Fogaça, L. J et al., 2020 - lower-limbs mean power
Ziyaiyan, A et al., 2023 - total reperirions
Fogaça, L. J et al., 2020 - upper-limbs peak power
Fogaça, L. J et al., 2020 - lower-limbs peak power
Caetano et al., 2023 - peak power
Caetano et al., 2023 - total reperirions
Omitted Effect
0.0
0.1
0.2
0.3
Hedges' g

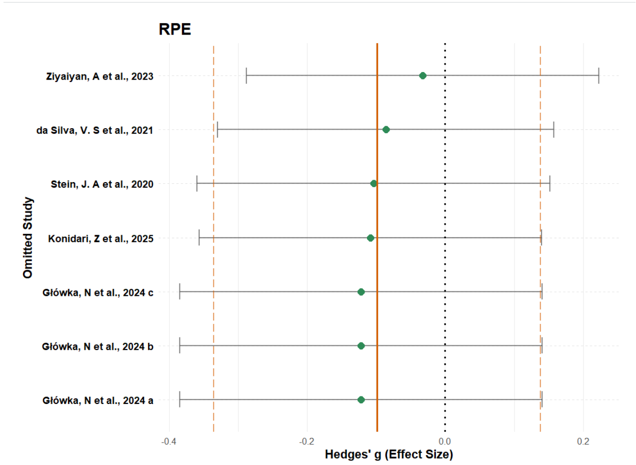
RPE
Ziyaiyan, A et al., 2023
da Silva, V. S et al., 2021
Stein, J. A et al., 2020
Konidari, Z et al., 2025
Główka, N et al., 2024 c
Główka, N et al., 2024 b
Główka, N et al., 2024 a
Omitted Study
-0.4
-0.2
0.0
0.2
Hedges' g (Effect Size)

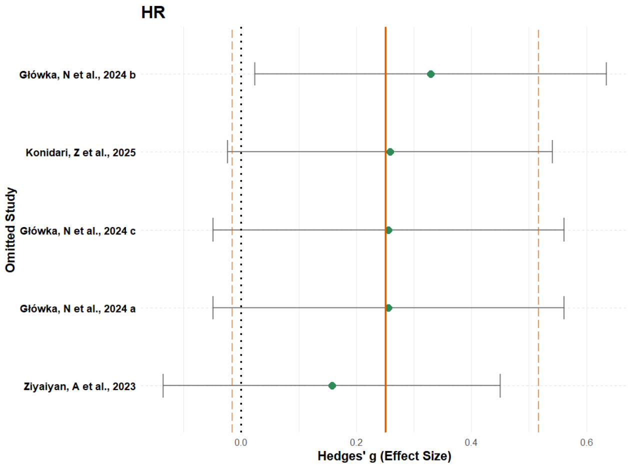
HR
Główka, N et al., 2024 b
Konidari, Z et al., 2025
Główka, N et al., 2024 c
Główka, N et al., 2024 a
Ziyaiyan, A et al., 2023
Omitted Study
0.0
0.2
0.4
0.6
Hedges' g (Effect Size)

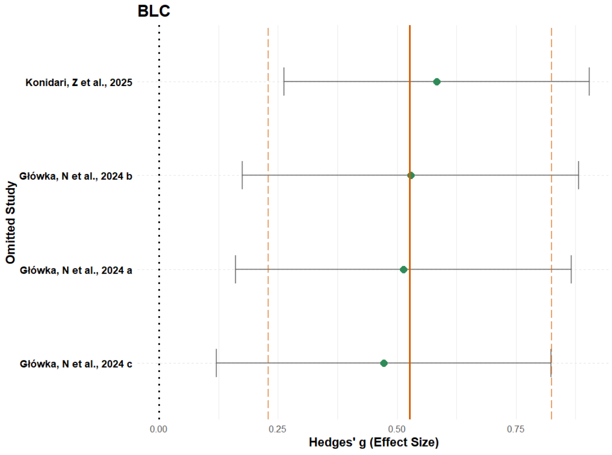
BLC
Konidari, Z et al., 2025
Główka, N et al., 2024 b
Główka, N et al., 2024 a
Główka, N et al., 2024 c
Omitted Study
0.00
0.25
0.50
0.75
Hedges' g (Effect Size)

Supplementary Material File S5 (Funnel plot)

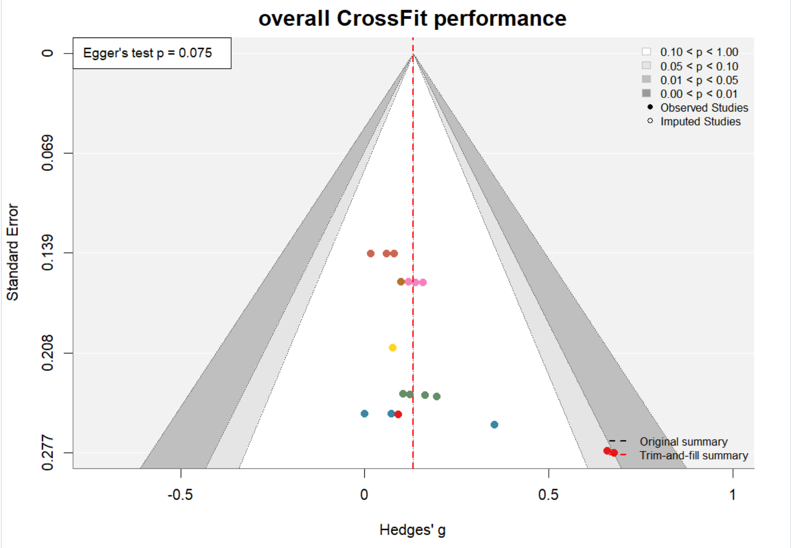

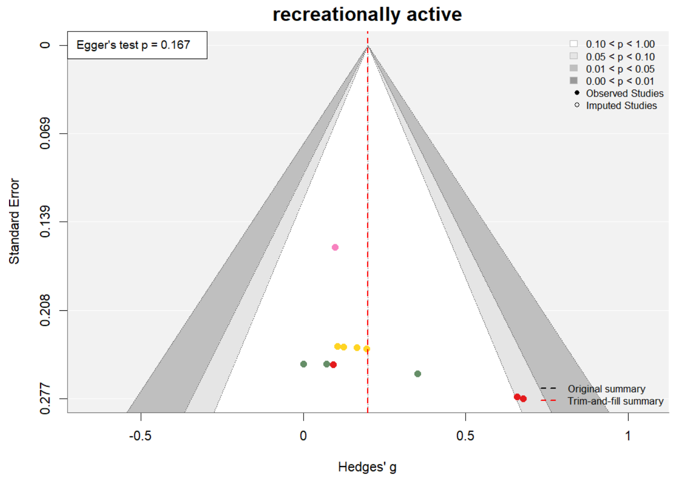

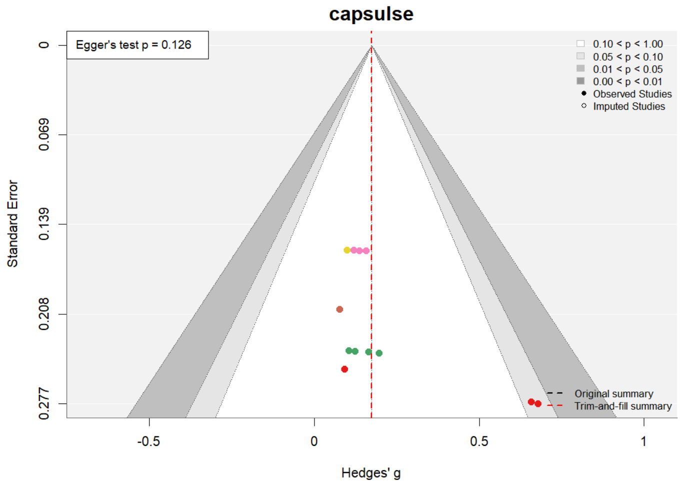

Supplementary File S6 (Manuscript Checklist)s

Section/topic	#	Checklist item	Reported on page #
**Can Blood Flow Restriction Amplify the Physiological and Performance Benefits of Interval Training in Male Intermittent-Sport Athletes? A Systematic Review and Meta-Analysis**			
Title	1	Identify the report as a systematic review, meta-analysis, or both.	Yes
**ABSTRACT**			
Structured summary	2	Provide a structured summary including, as applicable: background; objectives; data sources; study eligibility criteria, participants, and interventions; study appraisal and synthesis methods; results; conclusions and implications of key findings.	Yes
**INTRODUCTION**			
Rationale	3	Describe the rationale for the review in the context of what is already known.	Yes
Objectives	4	Provide an explicit statement of questions being addressed with reference to participants, interventions, comparisons, outcomes, and study design (PICOS).	Yes
**METHODS**			
Protocol and registration	5	Indicate if a review protocol exists, if and where it can be accessed (e.g., Web address), and, if available, provide registration information including registration number.	Yes
Eligibility criteria	6	Specify study characteristics (e.g., PICOS, length of follow-up) and report characteristics (e.g., years considered, language, publication status) used as criteria for eligibility, giving rationale.	Yes
Information sources	7	Describe all information sources (e.g., databases with dates of coverage) in the search.	Yes
Search	8	Present full electronic search strategy for at least one database, including any limits used, such that it could be repeated.	Yes
Study selection	9	State the process for selecting studies (i.e., screening, eligibility, included in systematic review, and, if applicable, included in the meta-analysis).	Yes
Data collection process	10	Describe method of data extraction from reports (e.g., piloted forms, independently, in duplicate) and any processes for obtaining and confirming data from investigators.	Yes
Data items	11	List and define all variables for which data were sought (e.g., PICOS) and any assumptions and simplifications made.	Yes
Risk of bias in individual studies	12	Describe methods used for assessing risk of bias of individual studies (including specification of whether this was done at the study), and how this information is to be used in any data synthesis.	Yes

Summary measures	13	State the principal summary measures (e.g., risk ratio, difference in means).	Yes
Synthesis of results	14	Describe the methods of handling data and combining results of studies, if done, including measures of consistency (e.g., I^2) for each meta-analysis.	Yes

**Section/topic**	**#**	**Checklist item**	**Reported on page #**
Risk of bias across studies	15	Specify any assessment of risk of bias that may affect the cumulative evidence (e.g., publication bias, selective reporting within studies).	Yes
Additional analyses	16	Describe methods of additional analyses (e.g., sensitivity or subgroup analyses, meta-regression), if done, indicating which were pre-specified.	Yes
**RESULTS**			
Study selection	17	Give numbers of studies screened, assessed for eligibility, and included in the review, with reasons for exclusions at each stage, ideally with a flow diagram.	Yes
Study characteristics	18	For each study, present characteristics for which data were extracted (e.g., study size, PICOS, follow-up period) and provide the citations.	Yes
Risk of bias within studies	19	Present data on risk of bias of each study and, if available, any outcome level assessment.	Yes
Results of individual studies	20	For all outcomes considered (benefits or harms), present, for each study: (a) simple summary data for each intervention group (b) effect estimates and confidence intervals, ideally with a forest plot.	Yes
Synthesis of results	21	Present results of each meta-analysis done, including confidence intervals and measures of consistency.	Yes
Risk of bias across studies	22	Present results of any assessment of risk of bias across studies.	Yes
Additional analysis	23	Give results of additional analyses, if done (e.g., sensitivity or subgroup analyses, meta-regression [see Item 16]).	Yes
**DISCUSSION**			
Summary of evidence	24	Summarize the main findings including the strength of evidence for each main outcome; consider their relevance to key groups (e.g., healthcare providers, users, and policy makers).	Yes
Limitations	25	Discuss limitations at study and outcome level (e.g., risk of bias), and at review-level (e.g., incomplete retrieval of identified research, reporting bias).	Yes
Conclusions	26	Provide a general interpretation of the results in the context of other evidence, and implications for future research.	Yes
**FUNDING**			

Funding	27	Describe sources of funding for the systematic review and other support (e.g., supply of data); role of funders for the systematic review.	Yes
